# Recent advancements in behavioral testing in rodents

**DOI:** 10.1016/j.mex.2021.101536

**Published:** 2021-09-28

**Authors:** Zeynep Sena AGIM USLU

**Affiliations:** Department of Neurology, University of Massachusetts Medical School, Worcester, Massachusetts 01605, USA

Behavioral testing in rodents is used to evaluate neurological traits and events, such as locomotor activity, depression-like behavior, socialization, memory, and many others. While behavior tests areare are indispensable part of neurological assessments, they also belong the group of the most challenging experimental setups. Researchers need to take many considerations into account in order to prevent unreproducible results and to decrease unnecessary labor. In this Article Collection, selected articles published in MethodsX were gathered to highlight the recent advancements in rodent behavioral testing all around the world and to assist scientific community in reaching reliable, reproducible, and validated methods.

Behavioral testing in animals is one of the most fundamental parts of assessing normal and diseasedconditions of human. However, succeeding reproducibility has been a growing problem not only among different labs, but also even different researchers of the very same lab. It is not uncommon that two blind researchers watch the same video and give different behavioral scores from another. In addition, post-hoc analysis of tens of videos from various numbers of subjects is inevitably a laborious work. In order to overcome such difficulties, Barbera *et al.* developed an automated capacitive touch sensing device that detects the number of direct social interactions to evaluate social behavior [1]. Abnormal social behavior, considered as an indication of neuropsychiatric disorders, could be assessed with the three-chamber social test where subject's interaction with novel vs familiar social environment is quantified. After the video is taken, blind researchers counted the number of physical touches of the subject with containers with or without another novel (or familiar) mouse. The setup described in the method article consists of capacitive touch sensors around containers so that the signal generated from poking/sniffing/touching/tasting could be recorded in an unbiased manner. Besides minimizing the time spent with video analysis and experimenter-to-experimenter variability, this automated, lowcost, and open-source solution has enabled real-time recordings that can be coupled with other real-time applications such as calcium activity recording, optogenetics, cerebral oxygen saturation measurement and many others, in freely moving animals.

Recent advancements in imaging technology have allowed imaging of and recording from single cells in the brain while performing behavioral test, simultaneously. Earlier this year, one of such applications that could be potentially coupled with the capacitive touch sensing device was also published in MethodsX. With a tiny microscope stereotaxically mounted on the skull-, neuronal activity in freely behaving animals could be recorded. Pritchard *et al.* described all steps with the crucial technical tweaks and details required to measure calcium activity within individual neurons, starting from the stereotaxic surgery and the placement of miniscope lenses to the behavioral testing and the immunofluorescent imaging [2]. The group used the same technique to reveal the underlying mechanism of association between activity of inhibitory and excitatory neurons in reticular thalamus and varicella zoster virus induced pain (shingles pain) [3]. Their findings suggest that this particular type of pain is caused by increased activity of GABAergic neurons and subsequent inhibition of excitatory neurons, which can be prevented by estradiol administration.

New methods or modifications to existing ones often come with new challenges. For instance, one limitation of the capacitive touch sensor device mentioned above was the false positive signals that were originated from the physical interaction between the containers and the cables [1]. To overcome this problem, the same group actually developed a motorized swivel system for behavioral and neural recordings in freely moving small rodents [4]. Via this arrangement, real-time measurements could be taken without encountering technical problems such as tangling and twisting of the cables. The upgraded system has several additional advantages. It is open source and cost-effective compared to others on the market. It is easy to manufacture (hardware needed can be either 3D printed or bought off-the-shelf) and subsequently scalable and modifiable according the individual experimental needs.

When it comes to behavioral testing, the source of variability is almost infinite. Yet researchers try to take into account and eliminate as many contributors as possible to obtain reliable and accurate results. For example, do motivational state, external stimuli or age change the behavior of rodents? Many of us answer this question in a heat beat: “Yes”. But how? A method article by researchers from the Michigan State University investigated this paradigm by using the Social versus Food Preference Test [5]. In their adaptation of the test, they used a three-chamber apparatus where there are two corrals with food or social (another rodent) at the opposite chambers and a neutral (empty) chamber in between. Wistar rats and C57BL/6 mice were recorded for 10 min, and parameters such as the time spent in each chamber and the time spent exploring each stimulus were quantified manually or using an automated tracking software. While the method article shared technical details of each step with the reader, it highlighted how the social over food preference was altered among different species, with deprivation, age and light vs dark phases. Together with their accompanying paper in Physiology and Behavior, these are some pivotal take-home massages [6]:

(1) When animals deprived from food prior to experiment, they favored the food over social stimulus. Interestingly, the change in preference was more profound in adolescents compared to adults. In addition, neither in rats nor mice the preference score was altered by social isolation.
(2) While the Wistar rats were more social preferring, the situation was the opposite for the C57BL/6 mice.
(3) No difference in time spent investigating the social and food stimuli was found between adolescent and adult C57BL/6 mice, but younger Wistar rats spent more time exploring compared to older ones.

I invite readers to dive deeper into these two articles to discover even more interesting findings. In the light of these findings, as it is stated in another independent article that also cited Reppucci and Veenema, hunger appears to be one of the top (if not the top) motivating forces changing behavior of species [7]. Hence, the conclusions drawn in the article should encourage researchers to elaborate more on the experimental set-up before designing their behavioral tests.

As Reppucci *et al.* discussed the effect of motivational state in behavior, Besosa and colleagues described a method where they used the motivational state (maternal instinct) to induce auditory associative learning [8]. Here, they used a T-maze where a female mouse and two pups are located in a nest area at the end of the long arm. After one pup was removed, an auditory signal was introduced from one of the small arms of the maze. Once the subject entered the correct arm (where sound came from), the pup was placed back again near the end of that arm. Quantification of the choice of the correct arm showed that subjects started learning to enter the arm with the sound to retrieve pups. This behavioral test presents itself as a core method for researchers who aim to investigate neurochemical and circuit mechanisms that mediate auditory associative learning, and their association with maternal behavior. Indeed, using this method, it has been showed that lactating mothers were more successful in learning to follow auditory cues to retrieve their pups, compared to cocaring female mice that co-habited in the same cage with pups and their mom [9]. This study is a very neat example of how this method was used to investigate the possible role of additional physiological and hormonal changes associated with the maternal state in sensory learning.

Besides the motivational state, age, sex, and species, the heterogeneity of the disease manifestation is one of the setbacks in maintaining the reproducibility of the scientific findings. Parkinson's disease (PD) is one the best examples of such a disorder with both non-motor and motor symptoms. According to widely accepted hypothesis, non-motor symptoms such as impairment in olfaction and abnormal bowel movements appear in years prior to diagnosis of motor symptoms. Therefore, it is crucial to evaluate both types of behaviors in potential PD models and therapeutics to examine the accurate representation and progression of the disease in line with human prognosis. In this context, the method article published by Soto-Rojas and colleagues carries great importance since it describes a sequential methodology to study both non-motor and motor manifestation of the disease [10]. Here, protocols for six behavioral tests that examined variety of sensorimotor alterations in PD rodent models were described: Open field test for locomotor activity, vibrissae test for sensorimotor alterations, olfactory test for loss of smell, uncoordinated gait test for balance and motor coordination, cylinder test for locomotor asymmetry, and lastly forced swim test for anxiety and depression-like behaviors. Thanks to the modifications listed detailed in the article, all these tests can be performed within a week and without the need for expensive or hard-to-acquire equipment, for improving the reproducibility regardless of wherever or by whoever the tests are performed. Although authors achieved validation of methodology using two unilateral PD models by injecting β-sitosterol-β-d-glucoside (BSSG) into substantia nigra or 6-hydroxydopamine into striatum, it is possible to use some these techniques for bilateral models with additional minor modifications. In their original research article in Behavioral Brain Research, they showed progression of the behavioral alterations accompanied by Lewy body-like inclusions and dopaminergic neuron loss by time after a single administration of BSSG [11]

Last but not least, we would like to mention another method article in MethodsX that proposes a diligent protocol to investigate the pharmacological intervention against memory consolidation and reconsolidation in the context of one of the most severe mental health problems of our time: Post-traumatic stress disorder (PTSD). It is a serious condition triggered by traumatic events in the past. As a part of cognitive behavioral therapy used for teaching how to change the behavioral pattern when the triggers remerge, medication can also be used to disrupt the fear memory. To study the pharmacological intervention, the Pavlovian fear conditioning is commonly used in the lab to model PTSD. Haider and colleagues provided step by step protocol to study different stages of fear memory in rats and to test various pharmacological agents that could potentially disrupt the adverse association [12]. The fundamental stages of the protocol consisted of habituation, training where an auditory signal was conditioned with a mild foot shock resulting in freezing, drug administration and testing sessions. Modifications in the protocol were introduced to examine different stages of memory recall. For instance, testing session was performed 2 hours (h) after the training session to assess short term memory, while 24 h waiting period was preferred for a long-term memory assessment. Another protocol pipeline introduced was chosen to examine reconsolidation. 24 h (recent memory) or 14 days (remote memory) after the training session, conditioned freezing behavior was reactivated followed by drug administration, and freezing times in testing sessions are quantified as an indication of intact short- and long-term memories. The protocol was also validated in their original research article in Life Sciences where atropine, a muscarinic acetylcholine receptor antagonist interfered with reconsolidation of old fear memories of Wistar rats [4], [13].

By gathering these seven method articles published in MethodsX during the years of 2020 and 2021 in an Article Collection, our goal was to help researchers reach the recent advancements and modifications in behavioral tests in rodents. As MethodsX, we are delighted to continue to publish methods and protocols from different scientific fields in an open-sourced and peer-reviewed platform, and to contribute to improvement of reproducibility among scientists all around the world.
